# The challenge of assessing impaired awareness of hypoglycaemia in diabetes in the era of continuous glucose monitoring: A narrative review of evidence and translation into clinical practice

**DOI:** 10.1111/dom.16284

**Published:** 2025-02-25

**Authors:** Simon A. Berry, Alexandros L. Liarakos, Vaios Koutroukas, Pratik Choudhary, Emma G. Wilmot, Ahmed Iqbal

**Affiliations:** ^1^ Division of Clinical Medicine, School of Medicine and Population Health University of Sheffield Sheffield UK; ^2^ Department of Diabetes and Endocrinology University Hospitals of Derby and Burton NHS Foundation Trust, Royal Derby Hospital Derby UK; ^3^ School of Medicine, Faculty of Medicine and Health Sciences University of Nottingham Nottingham UK; ^4^ Diabetes Research Centre University of Leicester Leicester UK

**Keywords:** continuous glucose monitoring, diabetes complications, hypoglycaemia, patient reported outcomes, type 1 diabetes, type 2 diabetes

## Abstract

Iatrogenic hypoglycaemia remains a major barrier in diabetes care. Over time, and with repeated hypoglycaemic episodes, the physiological responses to hypoglycaemia can become blunted, resulting in impaired awareness of hypoglycaemia (IAH). In IAH, the onset of cognitive dysfunction precedes the onset of autonomic symptoms, often preventing appropriate self‐treatment, thus increasing the frequency of severe hypoglycaemia (SH). Historically, IAH has been assessed with questionnaires, such as the Gold and Clarke scores, which were developed in the 1990s. A stepwise change in diabetes management in the last few decades has been the deployment of continuous glucose monitoring (CGM). CGM allows people with diabetes to set alarms that can warn them of hypoglycaemia or even impending hypoglycaemia, thus providing a degree of ‘technological’ awareness. This creates a challenge in assessing awareness status, as people may be alerted to low‐sensor glucose events before they experience any symptoms. CGM also allows the introduction of new measures of hypoglycaemia exposure such as time below range, which might complement traditional methods of risk assessment. These changes in the field prompt a need for reassessment of the measures of IAH. This narrative review evaluates the current epidemiology of SH and IAH, explores different measures of IAH, and evaluates the relationship between CGM metrics, IAH and SH. We conclude that a clinical approach involving traditional questionnaires, or newer updated alternatives such as the Hypo A‐Q awareness scale, combined with CGM metrics and clinical assessment of human factors is recommended in the absence of a clearly superior measure.

## 1 INTRODUCTION

More than a century from the discovery of insulin, iatrogenic hypoglycaemia remains a major barrier in diabetes care.^1^ In people with diabetes, hypoglycaemia is the consequence of the interaction between relative insulin excess from treatment and compromised physiological defences against falling plasma glucose.^1^ Hypoglycaemia is the critical limiting factor in intensive glycaemic control in diabetes, which is essential to prevent long term complications.^2^ Severe hypoglycaemia (SH) can occur if hypoglycaemia remains untreated, and is defined as hypoglycaemia leading to acute severe cognitive impairment requiring external assistance for recovery.^3^ SH can lead to death, with an estimated 8% of deaths of people with type 1 diabetes (T1D) followed‐up from childhood up to the age of 55 years of age attributed to hypoglycaemia.^4^ Mortality rates of people with T1D (pwT1D) at a later age of onset are less evidenced in the literature.

In normal physiology, the counterregulatory response to hypoglycaemia is activated at glucose levels of ~3.9 mmol/L, followed by the manifestation of autonomic symptoms at ~3.3 mmol/L and neuroglycopenic symptoms at ~2.8 mmol/L.^5^ Impaired awareness of hypoglycaemia (IAH) is the result of attenuated counterregulatory responses and reduced perception of symptoms to hypoglycaemia.^6^ Antecedent hypoglycaemia blunts the response to subsequent hypoglycaemia,^7^ leading to lower glycaemic thresholds for adrenaline release and initiation of autonomic symptoms than in people with normal awareness of hypoglycaemia (NAH).^8^ In IAH, the onset of cognitive dysfunction precedes the onset of symptoms, impairing the coordination and execution of self‐treatment, thus increasing the frequency of SH six‐fold in T1D and 17‐fold in insulin‐treated type 2 diabetes (T2D).^9^, ^10^ IAH exists on a spectrum, and awareness of hypoglycaemia and strength of counter‐regulation is affected day‐to‐day by factors such as the time of day, sleep and exercise.^11^, ^12^ IAH is also not a single entity, rather a clinical syndrome covering a heterogenous group of pathophysiological defects, all of which culminate in reduced awareness of hypoglycaemia symptoms. Described defects that contribute to counter‐regulatory impaired awareness include early loss of the glucagon response to hypoglycaemia,^13^ attenuated sympathoadrenal activity,^7^ altered central glucose sensing,^14^ and disruption of arousal and decision‐making centres of the brain.^15^ Increasingly IAH is seen in older adults with T1D with multiple comorbidities,^16^ reflecting two peaks of prevalence: the first in those with a recent onset of diabetes and a second in those with a longer duration of diabetes.^17^ IAH can be reversible and thus, identification of IAH is important to stratify a person with diabetes to the correct treatment.^18^, ^19^, ^20^, ^21^

**FIGURE 1:**
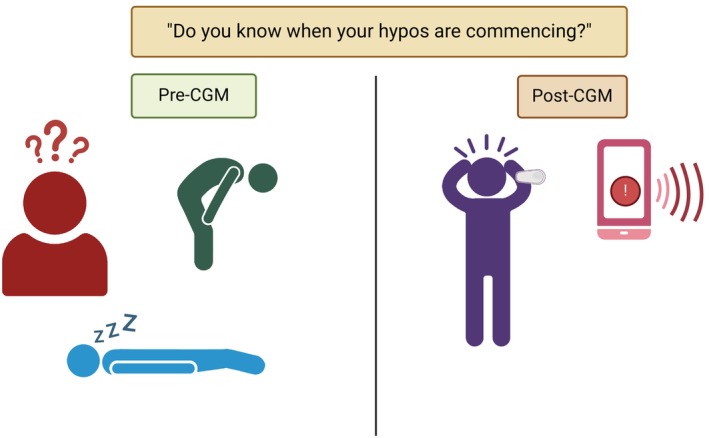
Illustration of the symptomatic presentation of hypoglycaemia in people with impaired awareness of hypoglycaemia before and after the introduction of Continuous Glucose Monitoring (CGM). Created with biorender.com.

Historically, IAH has been assessed with questionnaires developed in the 1990s. A GOLD or Clarke score ≥4 have been used as the diagnostic cut‐off.^22^, ^23^ Other scoring systems have since been developed.^24^, ^25^, ^26^ A stepwise change in diabetes management in the last few decades has been the ongoing development of continuous glucose monitoring (CGM).^27^ CGM can be set with alarms to warn a person with diabetes that the blood glucose level has dropped or is predicted to drop into the hypoglycaemic range.^28^ The person with diabetes may receive an alarm at a glucose level higher than when they typically experience symptoms and so may not be sure of what symptoms they get, blurring the lines between symptomatic awareness and ‘technological’ awareness. This may alter how pwT1D responds to questionnaires designed to assess awareness of hypoglycaemia (Figure 1). Furthermore, CGM allows the introduction of new measures of hypoglycaemia exposure such as time below range (TBR), which might complement traditional methods of risk assessment.^29^

These changes in the field prompt a need for reassessment of the measures of IAH. The aim of this narrative review is to identify the best tool or combination of tools to assess for IAH and the risk of SH in clinical practice based on research evidence. To achieve this, we evaluated the current prevalence of IAH and incidence of SH, explored the background of the different measures of IAH, detailed their components and reviewed both their historic and current evidence in terms of real‐world data and response to experimental hypoglycaemia. Finally, we evaluated the relationship between CGM metrics, IAH and SH. We used the keywords ‘severe hypoglycemia’, ‘impaired awareness of hypoglycemia’, ‘hypoglycemia awareness’ and ‘hypoglycemia unawareness,’ with synonyms, alone and in combination with each other and with keywords such as ‘prevalence’, ‘epidemiology’, ‘incidence’, ‘validation’ and ‘score OR questionnaire’ to retrieve available literature data from PubMed from inception until August 2024. The reference list from relevant papers was explored for further references and refined within the study group.

## 2 THE PREVALENCE OF IAH AND SEVERE HYPOGLYCAEMIA—HAS IT CHANGED?

**FIGURE 2:**
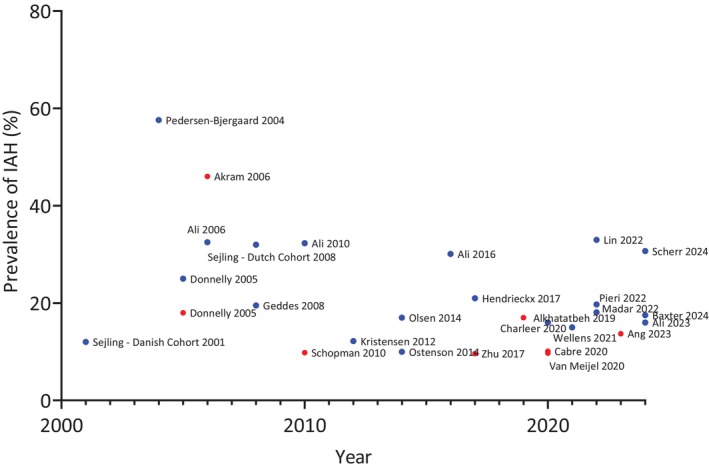
Prevalence of Impaired Awareness of Hypoglycaemia (IAH) in different studies over time in type 1 diabetes (blue) and type 2 diabetes (red). Please see Table S1 for data from individual studies.

The prevalence of IAH in T1D has historically been quoted as around 25%, based on retrospective case reviews from the 1980s and 1990s,^9^ whereas the prevalence of IAH in insulin‐treated T2D has been estimated to be around 10%.^10^ However, when individuals with T1D and T2D were matched for duration of treatment with insulin in one study, hypoglycaemia rates were comparable.^30^ Multiple studies over the last few decades have sought to update the estimated prevalence given improvements in clinical care and measures that reduce hypoglycaemia, such as structured education, newer insulin analogues, CGM and automated insulin delivery (AID) systems (Figure 2). Since 2020, studies have shown the estimated prevalence of IAH in T1D has decreased to between 15% and 18%. Exceptions were observational data from a unique patient population in a single tertiary centre, where the prevalence was estimated to be 33.3%, and data from the T1D Exchange, which estimated prevalence at 30.7% in a cohort of individuals historically shown to be highly engaged and more likely to achieve glycaemic targets.^31^, ^32^, ^33^, ^34^, ^35^ In insulin‐treated T2D, the estimated prevalence in studies since 2020 has been between 9.7% and 13.7%.^36^, ^37^, ^38^ There is considerable heterogeneity in the estimated prevalence between different studies. Reasons include the different methods used for diagnosis of IAH and the different baseline characteristics of studied populations.^9^ When studied in the same centre, the prevalence has been shown to have reduced over time.^33^ Of those in the cohort with data from all time points, the proportion of participants with persistent IAH dropped, supporting the idea that IAH is dynamic and reversible.^33^

The second question to be answered is whether the incidence of SH has changed over time. Evidence from randomized controlled trials (RCTs) (Table S2) indicates that CGM decreases the incidence of biochemical and severe hypoglycaemia when compared with self‐monitoring of blood glucose (SMBG) in pwT1D and a history of IAH and/or SH.^39^ Real‐world studies in Belgium, Norway and the United Kingdom demonstrated significant reductions in the incidence of SH after initiation of CGM, indicating that the benefits observed in RCTs are also applicable to real‐world use.^40^, ^41^, ^42^

**FIGURE 3:**
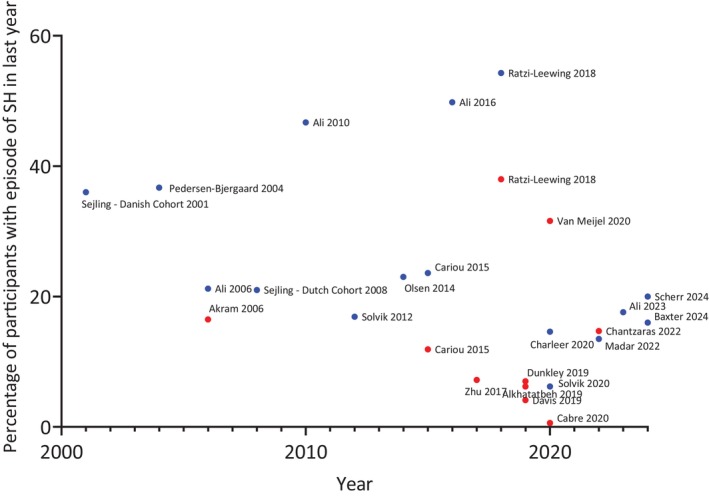
Proportion of participants with an episode of Severe Hypoglycaemia (SH) in last year – type 1 diabetes in blue, type 2 diabetes in red. Please see Table S1 for data from individual studies.

Therefore, given the increasing CGM use, one would expect observational data to show a marked reduction in the incidence of SH. Heterogeneity in the observational data makes it difficult to exactly quantify the incidence but recent studies demonstrate that between 6% and 20% of pwT1D reported an episode of SH in the last year (Figure 3).^32^, ^33^, ^35^, ^42^, ^43^ In people with insulin‐treated T2D, studies since 2020 have reported that between 0.6% and 31.6% of people had an episode of SH in the last year (Figure 3).^36^, ^37^, ^44^ One difficulty in drawing conclusions is the SH reporting methodology, because some studies report number of episodes per person year, whereas other studies report proportion of people with an episode of SH in the preceding year. These data are not easily comparable, as the incidence of SH per patient is not normally distributed but skewed towards more frequent episodes in high‐risk individuals.^45^ Furthermore, the reporting of SH is subject to considerable recall bias, particularly when recorded retrospectively.^46^ Other reasons for heterogeneity include different definitions of SH used between studies, and large inter‐regional differences.^46^, ^47^ Additionally, legal factors can influence participant reporting of episodes—there was a 55% drop in the reported incidence of SH after changes in driving laws in the European Union.^48^

Another important question is whether the risk of SH remains high with IAH when compared with people with NAH after CGM initiation. Similarly to older studies,^9^, ^10^ a recent cross‐sectional observational study demonstrated that an increased risk of SH in IAH persists in T1D when compared with those with intact awareness despite the use of CGM.^49^ IAH, as identified by the Gold, Clarke and Pedersen‐Bjergaard questionnaires, was associated with 6‐, 4.6‐ and 5.8‐fold increased risk of SH (*p* = 0.001, 0.004 and 0.013), respectively. In a further study by the same group, persistent SH despite CGM with alarms and AID systems was found to be influenced by participants' beliefs about prioritizing hyperglycaemia avoidance, suggesting that human factors may be responsible.^31^

Older studies, such as the DCCT trial, showed that low HbA1c was associated with higher rates of SH.^2^ However, multiple studies in the last decade have shown that this link has uncoupled. In the HAT study, there was no significant association between HbA1c and SH in both people with T1D and insulin‐treated T2D.^47^ Furthermore, both the GOLD randomized crossover trial and a post‐hoc analysis of the DIAMOND and HypoDE studies showed a dissociation between percent of time in hypoglycaemia and low HbA1c when CGM was used compared with SMBG.^50^, ^51^

In summary, the overall data indicate that the prevalence of IAH and incidence of SH in T1D is decreasing, but the incremental risk of SH in people with IAH over NAH remains a significant problem that needs addressing in clinical practice despite the use of new technologies such as CGM and AID systems.

## 3 MEASURES OF IMPAIRED AWARENESS OF HYPOGLYCAEMIA

### 3.1 Traditional measures of IAH

Several different tools and questionnaires have been developed to assess and diagnose IAH. The predominant tools that have been used clinically and in research are the Gold Score, Clarke Score, Pedersen‐Bjergaard Score, DAFNE tool, Hypo A‐Q Impaired Awareness Subscale and the HYPO score.^22^, ^23^, ^24^, ^25^, ^26^, ^52^ Modified versions of the Clarke Score and Pedersen‐Bjergaard Score now exist. They all ask different questions and vary in the time to complete, inclusion of past frequency of hypoglycaemia and methods of validation (Table 1). Validation can take multiple forms including real‐world comparison with rates of SH, discrimination analyses between IAH and normal awareness of hypoglycaemia (NAH) participants, and comparison with hyperinsulinaemic‐hypoglycaemic clamp study‐related metrics. In hyperinsulinaemic‐hypoglycaemic clamp studies, insulin and glucose infusions are used to lower the blood glucose to set targets to allow comparison between participant symptom scores, endogenous glucose production and counterregulatory hormone response.^53^ In real world, the depth and the duration of hypoglycaemic episodes can vary, whereas clamp studies allow for assessment of physiological responses in a controlled experimental setting.

**TABLE 1:** Comparison of questionnaire methods used for diagnosis of IAH.

	Gold	Minimally modified Clarke (MMCHS)	Updated Pedersen‐Bjergaard	DAFNE	HYPO	Hypo A‐Q impaired awareness subscale
Year of publication	1994	2012 (original 1995)	2022 (original 2003)	2002	2004	2016
No. of questions	1	8	1	1	4 weeks of data	5
Frequency of hypoglycaemia	No	Yes	No	No	Yes	No
Categories	IAH ≥4 NAH <4 (indeterminate = 3)	IAH ≥4 or 1× U NAH <4	IAH “Occasionally” or “Never” Intermediate “Usually” Normal “Always”	IAH <3 mmol/L NAH ≥3 mmol/L	No concern ≤423 Moderate issues 424–1046 Severe issues ≥1047	IAH ≥12 NAH <12
Advantages	Quick and simple question Used extensively for >20 years Used as Gold standard for many research trials Validated in hyper‐insulinaemic hypoglycaemic clamps Demonstrated improvement after intervention	Used extensively Used as Gold standard for many research trials Validated with high AUC in hyper‐insulinaemic hypoglycaemic clamps Validated pre‐ and post‐ intervention Demonstrated improvement after intervention	Quick and simple question Three categories incorporating spectrum of disease	Quick and simple question Cut‐off corresponds with Level 2 hypoglycaemia i.e. threshold of neuroglycopenic symptoms Demonstrated improvement after intervention	Useful research tool	Differentiates nocturnal from diurnal hypoglycaemia Validated against clamp, CGM and other IAH measures Compliant with FDA guidelines Validated in type 2 diabetes
Disadvantages	Dichotomous grading leads to inconsistencies when IAH is more borderline	Cut‐off not consistent with IHSG classifications of hypoglycaemia Inclusion of frequency of severe hypoglycaemia susceptible to improvements with new treatments Can lead to IAH diagnosis when awareness intact	Over‐estimates prevalence of IAH (addressed to some extent in updated version) Not validated alone in hyperinsulinaemic‐hypoglycaemic clamps	Not validated	Used predominantly as measure of hypoglycaemia severity Resource and time intensive	Tested on relatively few people compared with more established measures

Abbreviations: AUC, area under the curve; CGM, continuous glucose monitoring; IAH, impaired awareness of hypoglycaemia; IHSG, International Hypoglycemia Study Group; NAH, normal awareness of hypoglycaemia.

**FIGURE 4:**
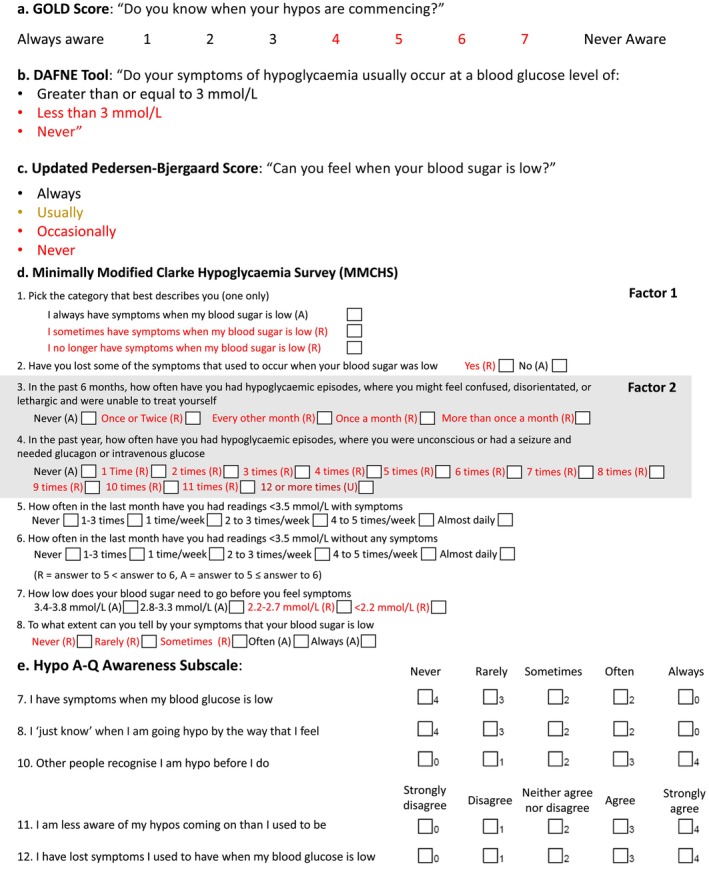
(A–C) Methods consisting of one question for assessing hypoglycaemia awareness status (A) Gold score (B) DAFNE tool (C) Updated Pedersen‐Bjergaard Score. Red denotes scores diagnostic of impaired awareness or loss of awareness in the Pedersen‐Bjergaard method, yellow denotes intermediate awareness.^22^, ^24^, ^61^ (D) Minimally Modified Clarke Questionnaire – ≥4 R Impaired Awareness, ≤3 aware, 1 U is unawareness. Factor 1, representing assessment of awareness, in white background. Factor 2, representing the assessment of hypoglycaemia exposure, in grey background.^23^ (E) Hypo A‐Q Awareness Subscale—Figure adapted from the version available at https://eprovide.mapi‐trust.org/instruments/hypoglycaemia‐awareness‐questionnaire. Accessed 21.10.2024.

The Gold score was developed in 1994 as a tool to categorize hypoglycaemia awareness in pwT1D, in a study prospectively investigating the frequency of hypoglycaemia in people with IAH.^22^ The score consists of a single question with participants asked to score between 1 and 7 on a Likert scale, with a score of ≥4 deemed as diagnostic of IAH (Figure 4A). In the original study, IAH was defined at a score of 4–7 as these scores were predominantly associated with neuroglycopenic rather than autonomic symptoms.

The Clarke score was developed in 1995 as a classification tool for hypoglycaemia awareness in people with insulin‐treated diabetes, in a study prospectively investigating the frequency and severity of hypoglycaemia^23^ (Figure 4D). Each question is answered with “R” for reduced awareness or “A” for normal awareness: IAH is defined as ≥4 “R” responses while NAH is ≤2 “R” responses. In the original questionnaire, if a person reports 12 or more SH episodes in the last year, they are automatically categorized as hypoglycaemia unaware, even if symptoms are intact. A minimally modified version of the Clarke hypoglycaemia survey (MMCHS) has been established, which updated question 4 to the current definition of SH, and reduced the blood glucose threshold for questions 5 and 6 to 3.5 mmol/L from 3.9 mmol/L.^54^ Importantly, the Clarke score incorporates two separate factors – factor 1 includes an assessment of hypoglycaemia awareness, whilst factor 2 questions assess hypoglycaemia exposure and risk of SH.^55^

The Pedersen‐Bjergaard score was developed to assess hypoglycaemia awareness in a study examining recall of SH in people with T1D.^26^ It consists of a single question and differs from the other scores by classifying people into three groups – normal awareness, impaired awareness and unawareness (Figure 4C). A criticism of the score was that it overestimated the prevalence of IAH. For example, in data by Geddes et al. (2007), the prevalence of IAH was 62.5% with Pedersen‐Bjergaard compared with 24% with the Gold score,^56^ and Gandhi et al. (2021) reported the prevalence of IAH at 61% with Pedersen‐Bjergaard compared with 19% with the Gold score.^57^ Meanwhile, Clarke and Gold scores consistently correlate with each other to a moderate‐to‐high degree.^56^, ^58^, ^59^ The original authors of the Pedersen‐Bjergaard score argue that the three categories give a more nuanced grading of IAH, with impaired awareness associated with a fivefold–sixfold increased rate of SH compared with NAH, and unawareness associated with a 10‐ to 20‐fold increased rate of SH.^60^ They have also argued that the method has been misunderstood and misused in other studies, with the unaware group better aligning with the impaired awareness groups from the Gold and Clarke scores.

In 2022, the Pedersen‐Bjergaard method was updated to change the category names to normal awareness, intermediate awareness and unawareness.^61^ When the Pedersen‐Bjergaard score is modified so that “occasionally” and “never” represent impaired awareness, a moderate or high correlation has been demonstrated with the Gold and Clarke score.^56^, ^57^, ^61^ The score has been validated in participants with clamp‐induced hypoglycaemia in combination with the Gold and Clarke scores but not in a head‐to‐head comparison.^62^

Both the Clarke and Gold Scores have been found to have a strong negative correlation with adrenaline and symptom response in hyperinsulinaemic‐hypoglycaemic clamp studies, strengthening their evidence base.^58^ However, in one study, 32% of participants were classified inconsistently by Clarke versus the Gold scores. The classification accuracy increased in both extremes of hypoglycaemia awareness.^58^ Recent analyses of the MMCHS have suggested that the diagnostic threshold for IAH may have changed over time.^55^ Improvements in the Clarke score after using CGM might be driven by reduction in SH rather than improvements in symptomatic awareness. The optimal operating point of the Clarke score based on a clamp study from 2023 would be ≥5 in contrast to the original cut‐off of 4.^63^ Alternatively, a score ≥2 comprising only questions 1, 2, 5/6, 7, 8 might be better at discriminating IAH from NAH than an overall score of ≥4.^55^

It is important to note that these scores of IAH have only been formally validated in pwT1D and not in T2D, yet multiple studies have used the Gold, Clarke and Pedersen‐Bjergaard scores to quantify IAH in people with T2D (pwT2D) (Figure 1). A survey‐based study by Ang et al. (2023) explored the use of Gold and Clarke scores in pwT2D on insulin and impaired awareness with good correlation with each other, suggesting a cut‐off ≥4 for Gold and ≥2.5 for the 5‐item MMCHS but no studies have validated them against clamp‐induced hypoglycaemia in T2D.^38^

### 3.2 DAFNE score

The DAFNE programme (2002) is the main structured educational intervention for pwT1D in the UK.^24^ As part of a 12‐month follow‐up study, hypoglycaemia awareness status was assessed based on the participants' response to what level of glucose they reported hypoglycaemic symptoms: those reporting symptoms at <3 mmol/L or absence of symptoms was categorized as having IAH, while those reporting symptoms at ≥3 mmol/L were categorized as having intact awareness of hypoglycaemia. This cut‐off corresponds with the threshold between Level 1 and Level 2 hypoglycaemia^3^ and blood concentrations at which cognitive impairment begins.^64^ In the study, those with IAH were 4 times more likely to have had an episode of SH in the last year than those without. The tool has the benefit of being a quick and simple question but no validation against other established measures of hypoglycaemia awareness or clamp‐induced hypoglycaemia has been completed.

### 3.3 HYPO score

The HYPO score was developed in 2004 as an objective measure of quantifying SH and glucose lability in people with diabetes who were being considered for islet transplantation.^52^ The score is composed of two parts. The first part includes the patients' self‐reported hypoglycaemic episodes requiring third‐party assistance, glucagon or ambulance attendance from the last year. The second part is a questionnaire about the number of documented hypoglycaemic episodes (<2.5 and 2.5–2.9 mmol/L). These data are collected over 4 weeks, with more points allocated for fewer or absent symptoms. The correlation with the history of previous‐year recalled SH and the 4‐week recorded HYPO score was statistically significant but weak (*r* = 0.335, *p* < 0.0001).^65^ In a clamp study aimed at validating clinical metrics of IAH, the HYPO score predicted the hypoglycaemia symptom response (AUC 0.79) but the Clarke score performed better (AUC 0.81).^66^ The HYPO score has been used since in research, predominantly as a measure of hypoglycaemic severity.^63^ However, it is a time‐consuming and resource‐intensive method for both the participant during the 4‐week monitoring period and for the clinician interpreting the raw data.^65^

### 3.4 Hypo A‐Q

The Hypoglycaemia Awareness Questionnaire (Hypo A‐Q) was developed in 2016 as a measure of hypoglycaemia awareness and problematic hypoglycaemia, with three different subscales reflecting hypoglycaemia awareness, symptom level and symptom frequency.^25^ The questionnaire was designed through exploratory and cognitive debriefing with pwT1D and IAH, modified by diabetologists and psychologists, and then underwent preliminary validation with a cohort of 120 adults with T1D, half of whom had IAH (Figure 4E). It is the only impaired awareness questionnaire that has been designed to meet US Food and Drug Administration (FDA) patient‐reported outcome measure (PROM) standards.^67^

Convergent validity was assessed by comparing the HypoA‐Q ‘impaired awareness’ subscale against Gold and Clarke questionnaire scores, showing good correlation (Gold *rs* = 0.75, *p* < 0.01; Clarke *rs* = 0.76, *p* < 0.01). Divergent validity was assessed by comparing the HypoA‐Q impaired awareness subscale against diabetes‐related distress (PAID total score), diabetes duration and HbA1c. Known‐groups validity evaluation demonstrated that the ‘hypoglycaemia awareness’ subscale (questions 7, 8, 10, 11, 12) and the questions related to the frequency of SH (questions 2a, 4b, 4c) distinguished between maintained and impaired awareness as defined by Gold score.

The above results were reproduced during a study that evaluated the validity of the Hypo A‐Q against hyperinsulinaemic‐hypoglycaemic clamp and CGM metrics.^63^ The ‘awareness subscale’ showed significant differences between participants with intact and those with impaired awareness, which correlated with differences in Clarke (*r* = 0.72, *p* < 0.001), HYPO score (*r* = 0.60, *p* < 0.001), CGM TBR (*r* = 0.53, *p* < 0.01), adrenaline (*r* = −0.68, *p* < 0.001) and autonomic symptom responses (*r* = −0.53, *p* < 0.05) between the two groups. The researchers further suggested a cut‐off threshold of ≥12 to identify people with impaired awareness based on correlation with a blunted adrenaline response during clamp‐induced hypoglycaemia. In addition, the HypoA‐Q score has uniquely undergone validation for assessing awareness of hypoglycaemia in pwT2D treated with insulin.^68^

A novel feature of the Hypo A‐Q score is the differentiation between nocturnal and diurnal hypoglycaemia. PwT1D involved in the design of the Hypo A‐Q questionnaire highlighted the differences in symptom presentation between day and night, driving its incorporation into the questionnaire.^25^ The Hypo A‐Q questionnaire, whilst novel, has only been validated in small numbers; further studies are needed. Consisting of only five questions, it is short enough to use as a clinical and research tool.

## 4 THE USE OF CGM TO PREDICT FUTURE SEVERE HYPOGLYCAEMIA

CGM has become an important part of diabetes management providing useful insights into glucose levels, glycaemic variability and hypoglycaemia. In clinical practice, TBR derived from CGM has become a key metric for the assessment of hypoglycaemia exposure and burden.^69^ The International Consensus on Time in Range recommends that <4% of time is spent <3.9 mmol/L and <1% of time is spent <3.0 mmol/L.^70^ An individual with several short hypoglycaemic episodes may have similar TBR to a person who has a single prolonged episode.^71^ However, a hypoglycaemic episode lasting under 15 minutes does not count towards TBR on CGM. This means that there is a risk of underappreciation of an individual's true hypoglycaemia exposure if TBR is used alone without a holistic view of daily events that could indicate potential risks and problematic behaviours. Human factors such as prioritizing hyperglycaemia avoidance, normalizing asymptomatic hypoglycaemia and minimizing hypoglycaemia concerns have been demonstrated to increase the risk of SH by driving self‐management decisions including excess or early insulin bolusing and insulin stacking.^72^

An important question is whether CGM metrics such as TBR can be used to assess IAH and/or predict future SH. Several studies have sought to answer the question of whether CGM metrics correlate with traditional measures of IAH such as the Clarke score. Vieira et al. (2022) showed that people with IAH, as assessed by Clarke score, had a significantly higher TBR and mean duration of hypoglycaemia compared with the other groups.^73^ The mean duration of hypoglycaemia was found to be an independent predictor of Clarke scores and a mean duration of hypoglycaemia ≥106.5 min showed 84.6% sensitivity and 64.4% specificity for IAH. A further cross‐sectional observational study of 99 pwT1D showed that IAH was associated with greater %CGM values <3.9 mmol/L and <3.0 mmol/L compared with NAH, and that this directly correlated to increasing Clarke scores. Importantly, IAH was related to more events lasting ≥20 minutes.^74^ Henriksen et al. (2018), conducted an observational study of 153 unselected pwT1D, and found that the higher the CGM hypoglycaemia events over 6 days, the higher the fraction of asymptomatic sensor‐detected hypoglycaemic episodes and the higher the risk of SH.^29^ In contrast, Choudhary and colleagues (2010) found no identifiable CGM differences in hypoglycaemia between pwT1D with and without IAH.^75^ A post‐hoc analysis of the DIAMOND and HypoDE trials showed that CGM‐derived low blood glucose index (LBGI) and %time <3.9 mmol/L (AUC 0.68–0.75) could significantly predict future SH, yet time <3.0 mmol/L demonstrated mixed results.^76^ Findings from post‐hoc analysis of the ABCD FreeStyle Libre Audit, including paired TBR, SH and Gold score data from 5029 pwT1D started on CGM, showed that TBR alone had a weak correlation with IAH and SH, only marginally better than random guessing (AUC 0.597 and 0.598 respectively).^77^ However, TBR cutoffs of 3.35% for IAH and 3.95% for SH, yielded high negative predictive values of 85% and 97% respectively, indicating that if TBR is below these cutoffs, there is a low chance of IAH or SH. Gold score alone had an AUC of 0.734 for predicting SH providing some evidence of its validity in the CGM era. Adding TBR to the Gold score did not confer much benefit, only marginally increasing the AUC to 0.747.

Studies have also addressed whether CGM‐detected hypoglycaemia contributes to reduced defences of SH by attenuating counterregulatory responses. Two recent hyperinsulinaemic‐hypoglycaemic clamp studies concluded that CGM‐derived TBR can predict impaired adrenaline response to hypoglycaemia in pwT1D.^78^, ^79^ Thomas et al. (2024) initiated 22 pwT1D on 14 days of blinded CGM, prior to a hyperinsulinaemic‐hypoglycaemic clamp. They found that CGM TBR was associated with a reduced adrenaline response (*r* = 0.555, *p* = 0.007), although it should be noted that the TBR values were higher than routinely seen in clinical practice: a third of the %TBR values were above 19%.^78^ In a study of 42 pwT1D comparing 1‐week blinded CGM to responses in clamp‐induced hypoglycaemia, a negative association between TBR and adrenaline response was also observed,^79^ particularly driven by level 2 hypoglycaemia. Another small study aimed to assess whether the combination of CGM metrics with traditional questionnaires could further increase accuracy.^66^ The authors demonstrated that a Clarke score with a cut‐off of ≥4 has excellent sensitivity in detecting absent symptom response in clamp‐induced hypoglycaemia of 100% but only had a specificity of 50%. A percentage time <3 mmol/L of ≥2.21% had an 89% sensitivity and 73% specificity in predicting a reduced autonomous symptom response. When the Clarke score and time <3 mmol/L of ≥2.21% were combined, the sensitivity remained at 89%, but the specificity increased further to 87%. When the Clarke score and time <3.9 mmol/L of ≥9.42% were combined, the sensitivity was 78% with a specificity of 93%. However, the %TBR required for these outcomes was higher than the 4% and 1% specified in the consensus guidelines.^70^

**FIGURE 5:**
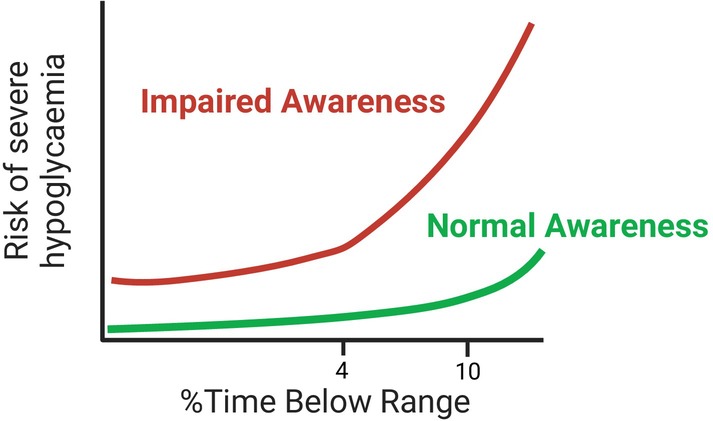
An illustration of risk of severe hypoglycaemia by awareness status and percentage time below range. Created with BioRender.com.

Overall, the data on whether CGM can be used to diagnose IAH is mixed requiring further elucidation. It appears there is scope to use TBR as a negative predictor to rule out a diagnosis of IAH. A better solution may be to combine TBR with IAH questionnaire scores, to increase the specificity of these already sensitive tests. Recent data has also shown how TBR can be used to predict future SH risk: TBR <4% confers a very low risk, irrespective of awareness status, yet only at much higher values (above 9%) does the TBR correlate with increased risk. The increased risk associated with higher TBR is amplified by impaired awareness as conceptually illustrated in Figure 5.

## 5 THE CHALLENGE OF ASSESSING IAH WITH CONCURRENT USE OF CGM

The use of CGM poses a number of challenges in how pwT1D and T2D answer the questionnaires, which predate the advent of CGM and AID systems. With threshold and predictive alarms, the person with diabetes may experience more hypoglycaemia via an alert or alarm at a glucose higher than when they would typically experience symptoms so they may not be sure of what symptoms they would experience. The structure of the questions in the IAH questionnaires fails to differentiate between technological awareness (sensor alarms) and physiological awareness (physical symptoms) of hypoglycaemia. Furthermore, technological advances have affected the clinical presentation of IAH: use of CGM reduces the frequency of SH without necessarily restoring the counter‐regulation response in people with IAH^80^ and AID systems have been shown to prevent nocturnal hypoglycaemic episodes.^81^ The Clarke score is particularly susceptible to change in SH frequency, even when symptomatic awareness has not improved.^55^ As a result, these tools may not be as suitable for discriminating impaired awareness in the modern CGM era, unless they are adapted or are further validated in future studies. Allaying some of these theoretical concerns, a few small studies have shown that the Clarke and Gold scores still correlate with symptom response in clamp studies and with frequency of SH, even with CGM use.^66^, ^77^ However, it is an area where there remains a lack of robust evidence.

Another key question is the relative importance of sensor‐detected hypoglycaemia (SDH) compared with person‐reported hypoglycaemia (PRH). The recently published HypoMETRICS observational study addressed this question in pwT1D and insulin‐treated T2D.^82^ The authors showed that SDH alone did not affect the daily functioning of participants whereas PRH (with or without SDH) was significantly associated with changes in daily functioning. Furthermore, it found that over half of SDH events (at thresholds of 3.9 and 3.0 mmol/L) were asymptomatic, even below 3 mmol/L in people deemed to have normal awareness, and conversely that many symptomatic hypoglycaemic episodes occur above 3.9 mmol/L.^83^ In a study using CGM sensors in those without diabetes, Shah et al. (2019) demonstrated that 28% of people without diabetes also experience SDH.^84^ There are also questions about the accuracy of CGM in the hypoglycaemic range: CGM has been shown to significantly overestimate the glucose level in the hypoglycaemic range, by 19% compared with arterialised glucose readings (*p* < 0.001), albeit with hyperinsulinaemia in an experimental hypoglycaemic clamp setting.^85^ However, CGM is a useful tool to identify glucose trends, and provides more comprehensive data than single capillary blood readings, which may not reflect the hypoglycaemic nadir nor provide data on before and after a hypoglycaemic event. Continuous data collection reduces the impact of recall bias when assessing prior hypoglycaemia. The HypoMETRICS app, which was used to survey self‐detected hypoglycaemia three times a day for the HypoMETRICS study, has also shown high completion rates and to be acceptable to participants, while allowing real‐time reporting of episodes, and therefore, might become a future research or even clinical tool to explore the real‐world impact of hypoglycaemia.^86^

## 6 DEVELOPING A CLINICAL APPROACH TO IMPAIRED AWARENESS OF HYPOGLYCAEMIA AND RISK OF SEVERE HYPOGLYCAEMIA

**FIGURE 6:**
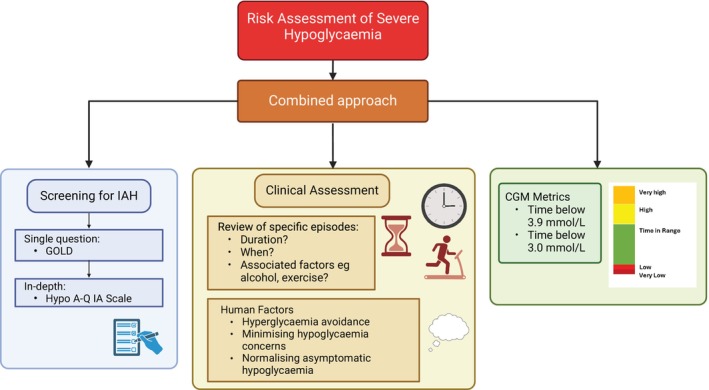
A clinical approach to risk assessment of severe hypoglycaemia. Created with BioRender.com. IAH—Impaired Awareness of Hypoglycaemia, CGM—Continuous Glucose Monitoring.

Evaluation of the current evidence has demonstrated the strengths and weaknesses of the tools available to the clinician assessing IAH and the risk of SH. Despite the limitations of the evidence including lack of complete validation in the presence of new technologies, and the unclear accuracy of CGM metrics to predict future SH, these tools remain useful in clinical practice. Current research demonstrates that combining different clinical metrics gives a more accurate risk assessment than one tool alone.^66^ Nuance is required when examining CGM data, beyond the headline metrics of the ambulatory glucose profile, to carefully examine all episodes of hypoglycaemia. These can correlate with problematic behaviours which should be sought via a broader holistic clinical assessment.^72^ We suggest a combination approach with equal weight given to multiple factors, in the absence of clear superiority of one measure over the others (Figure 6).

## 7 CONCLUSION AND QUESTIONS FOR THE FUTURE

Despite the arrival of new technologies such as CGM and AID, SH remains an important challenge in people living with diabetes. IAH continues to increase the risk of SH despite these new interventions. Where episodes of SH persist despite these treatments, clinical assessment is important to identify people who would benefit from structured education programmes, psychoeducational interventions and rescue treatment with islet cell transplants. The introduction of CGM and alarms indicating hypoglycaemia and impending hypoglycaemia has changed the assessment of SH by reducing its incidence. CGM metrics alone do not appear sufficiently accurate to predict future episodes of SH but are useful when used in combination with other measures. Questionnaire methods of diagnosing IAH have existed since the 1990s and are still used as the primary method of diagnosis. They have previously been validated in terms of prediction of SH, and prediction of symptom and counter‐regulatory response to experimental hypoglycaemia. In the era of modern CGM, the cut‐offs required for diagnosis and wording of questions are now under review. The different questionnaires and CGM metrics all use arbitrary thresholds, yet a spectrum of disease has been demonstrated in research and in clinical practice. Thus, questions remain over where thresholds are set. The Hypo A‐Q questionnaire offers a new measure of IAH with more careful construction and validation, and is the only questionnaire specifically designed to meet FDA PROM standards. A contemporary, well‐powered study, comparing all the methods described in this article in the presence of ubiquitous CGM use in people with diabetes with a spectrum of awareness would be beneficial to identify the most accurate tool or combination of tools, which are acceptable to patients and clinicians, to use in modern clinical practice. The CLEAR study (NCT06325202) is expected to address some of these challenges and further elucidate heterogeneity within IAH. A combination of measures, with holistic clinical assessment, is suggested in the absence of a clearly superior tool. Additionally, further research should be performed in pwT2D, where there have been fewer studies validating the current questionnaires.

## CONFLICT OF INTEREST STATEMENT

SAB has received research support from Dexcom and Tandem Diabetes Care. ALL has received speaker fees and/or support to attend conferences from Dexcom and Novo Nordisk and research support from the Association of British Clinical Diabetologists. VK has received support to attend conferences from KelCon GmbH and Eli Lilly. PC has received personal fees from Abbott, Dexcom, Insulet, Medtronic, Novo Nordisk, Lilly, Sanofi, Embecta, Vertex and Roche, and research support from Medtronic, Abbott, Dexcom and Novo Nordisk. EGW has received personal fees from Abbott, AstraZeneca, Dexcom, Eli Lilly, Embecta, Insulet, Medtronic, Novo Nordisk, Roche, Sanofi, Sinocare, and Ypsomed and research support from Abbott, Embecta, Insulet, Novo Nordisk, and Sanofi. AI has received research support from Dexcom and Abbott and consultancy fees from Eli Lilly, Boheringer Ingleheim, and AstraZeneca.

### PEER REVIEW

The peer review history for this article is available at https://www.webofscience.com/api/gateway/wos/peer-review/10.1111/dom.16284.

## Supporting information


**Table S1.** Observational studies with data on prevalence of Impaired Awareness of Hypoglycaemia (IAH) and incidence of severe hypoglycaemia (SH) included in Figures 2 and 3.

**Table S2.** Randomised controlled trials of technologies in adults with type 1 diabetes and impaired awareness of hypoglycaemia and/or severe hypoglycaemia.

## Data Availability

Data sharing not applicable to this article as no datasets were generated or analysed during the current study.
