# Supplementary material for: The challenge of assessing impaired awareness of hypoglycaemia in diabetes in the era of continuous glucose monitoring: A narrative review of evidence and translation into clinical practice
Source: Diabetes Obes Metab. 2025 Feb 25;27(5):2363–76. doi: 10.1111/dom.16284 (PMC11965031; doi:10.1111/dom.16284)
Supplement: Supplementary file 1 — Table S1. Observational studies with data on prevalence of Impaired Awareness of Hypoglycaemia (IAH) and incidence of severe hypoglycaemia (SH) included in Figures 2 and 3. [file DOM-27-2363-s001.docx]

**Supplementary Table 1.** Observational studies with data on prevalence of Impaired Awareness of Hypoglycaemia (IAH) and incidence of severe hypoglycaemia (SH) included in Figures 2 and 3

| **Study**  **(first author, year, design)** | **Population** | **Number of participants** | **Prevalence of IAH** | **Incidence of SH** |
| --- | --- | --- | --- | --- |
| Baxter, 2024 ^1^  Cross-sectional questionnaire study | T1D, single tertiary clinic in United Kingdom | 189 | 17.5% | 16% in last year |
| Ali, 2023 ^2^  Cross-sectional questionnaire study  Comparison with previous cross-sectional studies | T1D, single centre in the Netherlands | 488 in 2023 cohort | 2006: 32.5%  2010: 32.3%  2016: 30.1%  2023: 16% | 2006: 21.2% in last year  2010: 46.7% in last year  2016: 49.8% in last year  2023: 17.6% in last year |
| Geddes, 2008 ^3^  Cross-sectional questionnaire study | T1D, single centre in United Kingdom | 518 | 19.5% | N/A: data only reported as separate normal awareness and IAH |
| Olsen, 2014 ^4^  Cross-sectional questionnaire study | T1D, single centre in Norway | 440 | 17% | 23% in last year |
| Madar, 2022 ^5^  Cross-sectional study | T1D with duration ≥14 years,  BETTER registry from Quebec (Canada) | 1430 | 19.7% | T1D: 13.5% in last year |
| Khunti, 2016 ^6^  6-month retrospective and 4-week prospective study | T1D and insulin-treated T2D  Global HAT study | 27585, T1D: 8022, T2D: 19563 | - | T1D: 4.9 events/person-year, 14.4% in last 6 months  T2D: 8.9% in last 6 months, 2.5 events/person-year |
| Sejling, 2016 ^7^  Prospective observational study  Two Cohorts – one from Denmark with 12 years follow-up, one from Netherlands with 6.5 years follow-up | T1D  Danish Cohort 1999-2001 – 12-year follow-up  Dutch Cohort 2006-2008 – 6.5-year follow-up | Denmark: 269  Netherlands: 482 | Danish: 12% “unaware”  Dutch: 32% | Danish: 36% in first prospective year  Dutch: 21% in year before baseline (retrospective) |
| Cariou, 2015 ^8^ - DIALOG Study  Prospective (30-day) and retrospective observational study | T1D and insulin-treated T2D  Multicentre in France | 3048, T1D: 1915, T2D: 2509 | - | T1D: 23.6% in last year, 0.08 events/patient/month  T2D: 11.9% in last year, 0.05 events/patient/month |
| Hendrieckx, 2017 ^9^ – Diabetes MILES  Cross-sectional questionnaire study | T1D, Australia | 642 | 21% | T1D: 21% in last 6 months, 0.5 events per person in last 6 months |
| Lin, 2022 ^10^  Cross-sectional questionnaire study | T1D, single tertiary centre, USA | 289 | 33% | 25.6% in last 6 months |
| Wellens, 2021 ^11^  Cross-sectional study | T1D, two centres in the Netherlands | 509 | 15% | N/A: data only reported as separate normal awareness and IAH |
| Pedersen-Bjergaard, 2004 ^12^  Cross-sectional questionnaire study | T1D, Denmark and United Kingdom | 1076 | 57.6% | 36.7% in last year, 1.3 episodes/person-year |
| Donnelly, 2005 ^13^  Retrospective survey and prospective for one month | T1D and insulin-treated T2D  United Kingdom | 267, T1D 94, T2D 173 | T1D: 25%  T2D: 18% | T1D: 1.15 episodes/person-year  T2D: 0.35 episodes/person-year |
| Kristensen, 2012 ^14^  Cross-sectional questionnaire study | T1D  6 diabetes clinics in Denmark | 3861 | 12.2% “unaware” | 1.21 episodes/person-year |
| Ostenson, 2014 ^15^  Retrospective surveys repeated on 4 occasions | T1D and insulin-treated T2D  Austria, Denmark, Finland, Norway, Sweden, Switzerland and the Netherlands | 3827, T1D 1631, T2D 2196 | T1D: 10% “unaware”  T2D: 6-19% “unaware” | T1D: 0.7 episodes/person-year  T2D: 0.1-0.2 episodes/person-year |
| Solvik, 2024 ^16^  Cross-sectional and longitudinal registry-based study | T1D, Norwegian Diabetes Register  2012 and 2020 cohorts | 5746 in 2012  18984 in 2020 | N/A | 2012: 16.9% in last year  2020: 6.2% in last year |
| Pieri, 2022 ^17^  Observational study before and after FreeStyle Libre initiation | T1D, ABCD audit in United Kingdom | 4391 with paired gold data | 28.1% at baseline, 18.1% at follow-up | Baseline: 14.4% in last year, 140 per month in 14248 people  Follow-up: 4.7% in follow-up period (mean 7.6 months), 48 per month in 14248 people |
| Ratzki-Leewing, 2018 ^18^  Cross-sectional questionnaire-based study | T1D and insulin and/or insulin secretagogue-treated T2D, Canada | 552, T1D 94, T2DM 458 | N/A | T1D: 54.3% in last year, 2.4 episodes/person-year  T2D: 38.0% in last year, 2.5 episodes/person-year |
| Scherr, 2024 ^19^  Cross-sectional questionnaire-based study | T1D, from T1D Exchange Registry in USA | 2074 | 30.7% | 20% in last year |
| Charleer, 2020 ^20^, FUTURE study, prospective observational cohort study on CGM | T1D, 3 diabetes specialist centres in Belgium | 1913 | 16% | 14.6% in last year, 0.97 episodes/person-year |
| Muller, 2020 ^21^  Retrospective population-based study  Cohorts from 2006, 2011 and 2016 | T2D, including all treatments, using German health insurance data | 6.6m in 2006  7.9m in 2011  8.86m in 2016 | - | All treatments:   - 2006: 460 per 100,000 person-years - 2011: 490 per 100,000 person-years - 2016: 360 per 100,000 person-years |
| Akram, 2006 ^22^  Cross-sectional questionnaire-based study | Insulin-treated T2D, single centre in Denmark | 401 | 46% | 16.5% in last year, 0.44 episodes/person-year |
| Alkhatatbeh, 2019 ^23^  Cross-sectional questionnaire-based study | Insulin-treated T2D, Jordan | 388 | 17.01% | 6.19% in last year |
| Cabre, 2020 ^24^  Cross-sectional study | Insulin-treated T2D in 9 primary care centres, Spain | 157 | 10.1% | 0.636 episodes/person-year |
| Zhu, 2017 ^25^  Cross-sectional questionnaire-based study | Insulin-treated T2D, single tertiary centre in Singapore | 374 | 9.6% | 7.2% in last year |
| Chantzaras, 2022 ^26^  Prospective epidemiological study | Insulin-treated T2D, single tertiary centre in Greece | 817 | - | 14.7% in last year |
| Dunkley, 2019 ^27^  Prospective observational study | T2D (all treatments), 17 centres in United Kingdom | 325 participants | - | 6.6% in last year  (11.5% in insulin-treated T2D) |
| Van Meijel, 2020 ^28^  Cross-sectional observational study | Insulin-treated T2D, The Dutch Diabetes Pearl (established cohort) | 2350 | 9.7% | 31.6% in last year |
| Davis, 2019 ^29^  Longitudinal observational study | T2D, from the Fremantle Diabetes Study Phase II, Australia | 1551 | - | 4.1% in last year |
| Schopman, 2010 ^30^  Cross-sectional questionnaire-based study | Insulin-treated T2D | 122 | 9.8% | N/A: data only reported as separate normal awareness and IAH |
| Ang, 2023 ^31^  Cross-sectional questionnaire-based study | Insulin-treated T2D | 153 | 13.7% | N/A |
| Abbreviations: T1D – Type 1 Diabetes; T2D – Type 2 Diabetes; CGM – Continuous Glucose Monitoring; IAH- Impaired Awareness of Hypoglycaemia; SH – severe hypoglycaemia | | | | |

**Supplemental Reference List**

1. Baxter F, Baillie N, Dover A, Stimson RH, Gibb F, Forbes S. A cross-sectional questionnaire study: Impaired awareness of hypoglycaemia remains prevalent in adults with type 1 diabetes and is associated with the risk of severe hypoglycaemia. *PLOS ONE*. 2024-06-14 2024;19(6):e0297601. doi:10.1371/journal.pone.0297601

2. Ali N, Hamdaoui E, Soumia, Schouwenberg BJ, Tack CJ, De Galan BE. Fall in prevalence of impaired awareness of hypoglycaemia in individuals with type 1 diabetes. *Diabetic Medicine*. 2023-04-01 2023;40(4)doi:10.1111/dme.15042

3. Geddes J, Schopman JE, Zammitt NN, Frier BM. Prevalence of impaired awareness of hypoglycaemia in adults with Type 1 diabetes. *Diabetic Medicine*. 2008-04-01 2008;25(4):501-504. doi:10.1111/j.1464-5491.2008.02413.x

4. Olsen SE, Åsvold BO, Frier BM, Aune SE, Hansen LI, Bjørgaas MR. Hypoglycaemia symptoms and impaired awareness of hypoglycaemia in adults with Type 1 diabetes: the association with diabetes duration. *Diabetic Medicine*. 2014-10-01 2014;31(10):1210-1217. doi:10.1111/dme.12496

5. Madar H, Wu Z, Bandini A, et al. Influence of severe hypoglycemia definition wording on reported prevalence in adults and adolescents with type 1 diabetes: a cross-sectional analysis from the BETTER patient-engagement registry analysis. *Acta Diabetologica*. 2022-10-17 2022;60(1):93-100. doi:10.1007/s00592-022-01987-9

6. Khunti K, Alsifri S, Aronson R, et al. Rates and predictors of hypoglycaemia in 27 585 people from 24 countries with insulin‐treated type 1 and type 2 diabetes: the global HAT study. *Diabetes, Obesity and Metabolism*. 2016-09-01 2016;18(9):907-915. doi:10.1111/dom.12689

7. Sejling AS, Schouwenberg B, Færch LH, Thorsteinsson B, De Galan BE, Pedersen‐Bjergaard U. Association between hypoglycaemia and impaired hypoglycaemia awareness and mortality in people with Type 1 diabetes mellitus. *Diabetic Medicine*. 2016-01-01 2016;33(1):77-83. doi:10.1111/dme.12810

8. Cariou B, Fontaine P, Eschwege E, et al. Frequency and predictors of confirmed hypoglycaemia in type 1 and insulin-treated type 2 diabetes mellitus patients in a real-life setting: Results from the DIALOG study. *Diabetes & metabolism*. 2015;41(2):116-125. doi:10.1016/j.diabet.2014.10.007

info:doi/10.1016/j.diabet.2014.10.007

9. Hendrieckx C, Hagger V, Jenkins A, Skinner TC, Pouwer F, Speight J. Severe hypoglycemia, impaired awareness of hypoglycemia, and self-monitoring in adults with type 1 diabetes: Results from Diabetes MILES—Australia. *Journal of diabetes and its complications*. 2017;31(3):577-582. doi:10.1016/j.jdiacomp.2016.11.013

info:doi/10.1016/j.jdiacomp.2016.11.013

10. Lin YK, Richardson CR, Dobrin I, et al. Beliefs Around Hypoglycemia and Their Impacts on Hypoglycemia Outcomes in Individuals with Type 1 Diabetes and High Risks for Hypoglycemia Despite Using Advanced Diabetes Technologies. *Diabetes Care*. 2022-03-01 2022;45(3):520-528. doi:10.2337/dc21-1285

11. Wellens MJ, Vollenbrock CE, Dekker P, et al. Residual C-peptide secretion and hypoglycemia awareness in people with type 1 diabetes. *BMJ Open Diabetes Research & Care*. 2021-09-01 2021;9(1):e002288. doi:10.1136/bmjdrc-2021-002288

12. Pedersen‐Bjergaard U, Pramming S, Heller SR, et al. Severe hypoglycaemia in 1076 adult patients with type 1 diabetes: influence of risk markers and selection. *Diabetes/metabolism research and reviews*. 2004;20(6):479-486. doi:10.1002/dmrr.482

info:doi/10.1002/dmrr.482

13. Donnelly LA, Morris AD, Frier BM, et al. Frequency and predictors of hypoglycaemia in Type 1 and insulin‐treated Type 2 diabetes: a population‐based study. *Diabetic medicine : a journal of the British Diabetic Association*. 2005;22(6):749-755. doi:10.1111/j.1464-5491.2005.01501.x

info:doi/10.1111/j.1464-5491.2005.01501.x

14. Kristensen PL, Hansen LS, Jespersen MJ, et al. Insulin analogues and severe hypoglycaemia in type 1 diabetes. *Diabetes research and clinical practice*. 2012;96(1):17-23. doi:10.1016/j.diabres.2011.10.046

info:doi/10.1016/j.diabres.2011.10.046

15. Östenson CG, Geelhoed‐Duijvestijn P, Lahtela J, Weitgasser R, Markert Jensen M, Pedersen‐Bjergaard U. Self‐reported non‐severe hypoglycaemic events in Europe. *Diabetic Medicine*. 2014-01-01 2014;31(1):92-101. doi:10.1111/dme.12261

16. Sølvik UØ, Cooper JG, Løvaas KF, et al. A register‐based study describing time trends in risk factor control and serious hypoglycaemia together with the effects of starting continuous glucose monitoring in people with type 1 diabetes in Norway. *Diabetic Medicine*. 2024-07-01 2024;41(7)doi:10.1111/dme.15335

17. Pieri B, Deshmukh H, Wilmot EG, et al. Impaired awareness of hypoglycaemia: Prevalence and associated factors before and after FreeStyle Libre use in the Association of British Clinical Diabetologists audit. *Diabetes, Obesity and Metabolism*. 2023-01-01 2023;25(1):302-305. doi:10.1111/dom.14841

18. Ratzki-Leewing A, Harris SB, Mequanint S, et al. Real-world crude incidence of hypoglycemia in adults with diabetes: Results of the InHypo-DM Study, Canada. *BMJ Open Diabetes Research & Care*. 2018-04-01 2018;6(1):e000503. doi:10.1136/bmjdrc-2017-000503

19. Sherr JL, Laffel LM, Liu J, et al. Severe Hypoglycemia and Impaired Awareness of Hypoglycemia Persist in People With Type 1 Diabetes Despite Use of Diabetes Technology: Results From a Cross-sectional Survey. *Diabetes Care*. 2024-06-01 2024;47(6):941-947. doi:10.2337/dc23-1765

20. Charleer S, De Block C, Van Huffel L, et al. Quality of Life and Glucose Control After 1 Year of Nationwide Reimbursement of Intermittently Scanned Continuous Glucose Monitoring in Adults Living With Type 1 Diabetes (FUTURE): A Prospective Observational Real-World Cohort Study. *Diabetes Care*. 2020-02-01 2020;43(2):389-397. doi:10.2337/dc19-1610

21. Müller N, Lehmann T, Klöss A, Günster C, Kloos C, Müller UA. Changes in incidence of severe hypoglycaemia in people with type 2 diabetes from 2006 to 2016: analysis based on health insurance data in Germany considering the anti‐hyperglycaemic medication. *Diabetic Medicine*. 2020-08-01 2020;37(8):1326-1332. doi:10.1111/dme.14294

22. Akram K, Pedersen‐Bjergaard U, Carstensen B, Borch‐Johnsen K, Thorsteinsson B. Frequency and risk factors of severe hypoglycaemia in insulin‐treated Type 2 diabetes: a cross‐sectional survey. *Diabetic medicine : a journal of the British Diabetic Association*. 2006;23(7):750-756. doi:10.1111/j.1464-5491.2006.01880.x

info:doi/10.1111/j.1464-5491.2006.01880.x

23. Alkhatatbeh MJ, Abdalqader NA, Alqudah MAY. Impaired Awareness of Hypoglycaemia in Insulin-treated Type 2 Diabetes Mellitus. *Current Diabetes Reviews*. 2019;15(5):407-413. doi:10.2174/1573399814666180806144937

info:doi/10.2174/1573399814666180806144937

24. Cabré C, Colungo C, Vinagre I, Jansà M, Conget I. Frequency and awareness of hypoglycemia in patients with Type 2 Diabetes treated with two or more insulin injections in primary care outpatient clinics. *Primary care diabetes*. 2020;14(2):168-172. doi:10.1016/j.pcd.2019.08.001

info:doi/10.1016/j.pcd.2019.08.001

25. Zhu L, Ang LC, Tan WB, et al. A study to evaluate the prevalence of impaired awareness of hypoglycaemia in adults with type 2 diabetes in outpatient clinic in a tertiary care centre in Singapore. *Therapeutic Advances in Endocrinology and Metabolism*. 2017-05-01 2017;8(5):69-74. doi:10.1177/2042018817707422

26. Chantzaras A, Yfantopoulos J. Evaluating the Incidence and Risk Factors Associated With Mild and Severe Hypoglycemia in Insulin-Treated Type 2 Diabetes. *Value in health regional issues*. 2022;30:9-17. doi:10.1016/j.vhri.2021.10.005

info:doi/10.1016/j.vhri.2021.10.005

27. Dunkley AJ, Fitzpatrick C, Gray LJ, et al. Incidence and severity of hypoglycaemia in type 2 diabetes by treatment regimen: A UK multisite 12‐month prospective observational study. *Diabetes, Obesity and Metabolism*. 2019-07-01 2019;21(7):1585-1595. doi:10.1111/dom.13690

28. Van Meijel LA, De Vegt F, Abbink EJ, et al. High prevalence of impaired awareness of hypoglycemia and severe hypoglycemia among people with insulin-treated type 2 diabetes: The Dutch Diabetes Pearl Cohort. *BMJ Open Diabetes Research & Care*. 2020-02-01 2020;8(1):e000935. doi:10.1136/bmjdrc-2019-000935

29. Davis TME, Bruce DG, Finn J, Curtis BH, Barraclough H, Davis WA. Temporal changes in the incidence and predictors of severe hypoglycaemia in type 2 diabetes: The Fremantle Diabetes Study. *Diabetes, Obesity and Metabolism*. 2019-03-01 2019;21(3):648-657. doi:10.1111/dom.13568

30. Schopman JE, Geddes J, Frier BM. Prevalence of impaired awareness of hypoglycaemia and frequency of hypoglycaemia in insulin-treated Type 2 diabetes. *Diabetes research and clinical practice*. 2010;87(1):64-68. doi:10.1016/j.diabres.2009.10.013

info:doi/10.1016/j.diabres.2009.10.013

31. Ang LC, Bee YM, Goh S-Y, Teh MM. New insights into the currently available questionnaire for assessing impaired awareness of hypoglycaemia (IAH) among insulin-treated type 2 diabetes- A key risk factor for hypoglycaemia. *Diabetes epidemiology and management*. 2023;10:100136. doi:10.1016/j.deman.2023.100136

info:doi/10.1016/j.deman.2023.100136
