# Supplementary material for: The challenge of assessing impaired awareness of hypoglycaemia in diabetes in the era of continuous glucose monitoring: A narrative review of evidence and translation into clinical practice
Source: Diabetes Obes Metab. 2025 Feb 25;27(5):2363–76. doi: 10.1111/dom.16284 (PMC11965031; doi:10.1111/dom.16284)
Supplement: Supplementary file 2 — Table S2. Randomised controlled trials of technologies in adults with type 1 diabetes and impaired awareness of hypoglycaemia and/or severe hypoglycaemia. [file DOM-27-2363-s002.docx]

**Supplemental Table 2.** Randomised controlled trials of technologies in adults with type 1 diabetes and impaired awareness of hypoglycaemia and/or severe hypoglycaemia

| **Study**  **(first author, year, name of trial, design, intervention duration)** | **Inclusion criteria** | **Number of participants**  **intervention/control** | **Mean age (years)**  **intervention/ control** | **Intervention (type of CGM) vs comparator** | **Primary outcome**  **& Results** |
| --- | --- | --- | --- | --- | --- |
| **Renard et al., 2023**  **iDCL** (1)  2-arm RCT  12 weeks  (with optional 12-week extension phase) | CSII  Clarke Hypoglycaemia Perception Awareness scale score >3 and/or SH during the past 6 months confirmed by TBR (<3.9 mmol/L) ≥5% for 2 weeks | 49/23 | 47/47 | AID  vs  CSII + CGM | Primary outcome: TBR change from baseline  AID vs control was associated with a reduction in TBR by 3.7% (95% CI -4.8, -2.6; p < 0.001), an increase in TIR by 8.6% (95% CI 5.2, 12.1; p < 0.001), and a decrease in TAR of 5.3% (95% CI -8.7, -1.8; p = 0.004).  During the 12-week extension phase, the effects of AID were sustained in the AID group and reproduced in the control group. |
| **Uduku et al., 2023** (2)  2-arm RCT  12 weeks | MDI  History of SH requiring emergency medical services | 15/8 | 40/48 | rtCGM  vs  SMBG | Primary outcome: difference between groups in % TBR (≤3.0 mmol/L)  The rtCGM group had a significantly larger reduction in %TBR (rtCGM -0.16%; 95% CI -1.23%, 0.01%] vs SMBG 1.58%; 95% CI 0.41, 3.48; p = 0.03) |
| **Heinemann et al., 2018**  **HypoDE** (3)  2-arm RCT  26 weeks | MDI  History of IAH or SH in past year | 75/66 |  | rtCGM  vs  SMBG | Primary outcome: baseline-adjusted number of hypoglycaemic events (defined as glucose ≤3.0 mmol/L for ≥20 min) during the follow-up phase  Incidence of hypoglycaemic events decreased by 72% for participants in the rtCGM group (incidence rate ratio 0.28 [95% CI 0.20-0.39], p <0.0001). |
| **van Beers., 2016**  **IN CONTROL** (4)  2-arm crossover trial  32 weeks | MDI or CSII  History of IAH | 26/26 |  | rtCGM, followed by SMBG  vs  SMBG, followed by rtCGM | Primary outcome: mean difference in % TIR (4-10 mmol/L)  TIR was higher during rtCGM than during SMBG: 65.0% (95% CI 62.8-67.3) vs 55.4% (53.1-57.7; mean difference 9.6%; 95% CI 8·0-11·2; p<0.0001) |
| **Reddy et al., 2018**  **I-HART** (5)  2-arm RCT  8 weeks | MDI  History of IAH or SH in past year | 40 | 50 | rtCGM  vs  isCGM | Primary outcome: difference in TBR (<3.3 mmol/L)  rtCGM vs isCGM significantly reduced TBR at the end-point (2.4% vs 6.8%; median between group difference -4.3%, p = 0.006). |
| **Little et al., 2014 & 2018**  **HypoCOMPass** (6, 7)  2 x 2 factorial RCT  24 weeks | MDI or CSII  History of IAH | 96 |  | CSII vs MDI  and  rtCGM vs SMBG | Primary outcome: between-intervention difference in 24-week hypoglycaemia awareness (Gold score).  Hypoglycaemia awareness improved (5.1 ± 1.1 to 4.1 ± 1.6; P = 0.0001) with decreased SH (8.9 ± 13.4 to 0.8 ± 1.8 episodes/patient-year; p = 0.0001).  At 24 weeks, there was no significant difference in awareness comparing CSII with MDI (4.1 ± 1.6 vs. 4.2 ± 1.7; difference 0.1; 95% CI -0.6 to 0.8) and rtCGM with SMBG (4.3 ± 1.6 vs. 4.0 ± 1.7; difference -0.3; 95% CI -1.0 to 0.4).  **Two-year follow-up results**  Improvement in hypoglycaemia awareness was sustained (Gold score at baseline 5.1 ± 1.1 vs. 24 months 3.7 ± 1.9; p < 0.0001).  Severe hypoglycaemia rate reduced from 8.9 ± 12.8 episodes/person-year over the 12 months pre-study to 0.4 ± 0.8 over 24 months (p < 0.0001).  HbA1c improved (baseline 8.2 ± 3.2% [66 ± 12 mmol/mol] vs. 24 months 7.7 ± 3.1% [61 ± 10 mmol/mol]; p = 0.003). |
| AID: Automated Insulin Delivery; CGM: Continuous Glucose Monitoring; CI: Confidence Interval; CSII: Continuous Subcutaneous Insulin Infusion; HbA1c: Haemoglobin A1c; IAH: impaired awareness of hypoglycaemia; isCGM: intermittently scanned Continuous Glucose Monitoring; MDI: Multiple Daily Insulin injections; RCT: Randomised Controlled Trial; rtCGM: real time Continuous Glucose Monitoring; SH: severe hypoglycaemia; SMBG: Self-Monitoring of Blood Glucose; TAR: Time Above Glucose Range; TBR: Time Below Glucose Range; TIR: Time In Range | | | | | |

**Supplemental References**

1. Renard E, Joubert M, Villard O, Dreves B, Reznik Y, Farret A, et al. Safety and Efficacy of Sustained Automated Insulin Delivery Compared With Sensor and Pump Therapy in Adults With Type 1 Diabetes at High Risk for Hypoglycemia: A Randomized Controlled Trial. Diabetes Care. 2023;46(12):2180-7.

2. Uduku C, Pendolino V, Jugnee N, Oliver N, Fothergill R, Reddy M. Real‐time continuous glucose monitoring immediately after severe hypoglycaemia requiring emergency medical services: A randomised controlled trial. Diabetic Medicine. 2023;40(7).

3. Heinemann L, Freckmann G, Ehrmann D, Faber-Heinemann G, Guerra S, Waldenmaier D, et al. Real-time continuous glucose monitoring in adults with type 1 diabetes and impaired hypoglycaemia awareness or severe hypoglycaemia treated with multiple daily insulin injections (HypoDE): a multicentre, randomised controlled trial. The lancet. 2018;391(10128):1367-77.

4. van Beers CAJ, DeVries JH, Kleijer SJ, Smits MM, Geelhoed-Duijvestijn PH, Kramer MHH, et al. Continuous glucose monitoring for patients with type 1 diabetes and impaired awareness of hypoglycaemia (IN CONTROL): a randomised, open-label, crossover trial. The lancet. 2016;4(11):893-902.

5. Reddy M, Jugnee N, El Laboudi A, Spanudakis E, Anantharaja S, Oliver N. A randomized controlled pilot study of continuous glucose monitoring and flash glucose monitoring in people with Type 1 diabetes and impaired awareness of hypoglycaemia. Diabetic Medicine. 2018;35(4):483-90.

6. Little SA, Leelarathna L, Walkinshaw E, Tan HK, Chapple O, Lubina-Solomon A, et al. Recovery of Hypoglycemia Awareness in Long-standing Type 1 Diabetes: A Multicenter 2 × 2 Factorial Randomized Controlled Trial Comparing Insulin Pump With Multiple Daily Injections and Continuous With Conventional Glucose Self-monitoring (HypoCOMPaSS). Diabetes Care. 2014;37(8):2114-22.

7. Little SA, Speight J, Leelarathna L, Walkinshaw E, Tan HK, Bowes A, et al. Sustained Reduction in Severe Hypoglycemia in Adults With Type 1 Diabetes Complicated by Impaired Awareness of Hypoglycemia: Two-Year Follow-up in the HypoCOMPaSS Randomized Clinical Trial. Diabetes Care. 2018;41(8):1600-7.
